# Pilot Study Exploring if an Augmented Reality NeedleTrainer Device Improves Novice Performance of a Simulated Central Venous Catheter Insertion on a Phantom

**DOI:** 10.7759/cureus.40197

**Published:** 2023-06-09

**Authors:** Annie Evans, Sean Shevlin, David Burckett-St.Laurent, James Bowness, Rachel J Kearns, Alan MacFarlane

**Affiliations:** 1 General Surgery, West of Scotland Foundation Deanery, Glasgow Royal Infirmary, Glasgow, GBR; 2 Anaesthesia, Belfast Health and Social Care Trust, Belfast, GBR; 3 Anaesthesia, Royal Gwent Hospital, Newport, GBR; 4 Anaesthesia, Aneurin Bevan University Health Board, Newport, GBR; 5 Anaesthesia, Princess Royal Maternity Hospital, Glasgow, GBR; 6 Anaesthesia, Glasgow Royal Infirmary, Glasgow, GBR

**Keywords:** ultrasound, education, central venous catheter, augmented reality, simulation

## Abstract

Introduction

Needle insertion and visualisation skills needed for ultrasound (US)-guided procedures can be challenging to acquire. The novel NeedleTrainer device superimposes a digital holographic needle on a real-time US image display without puncturing a surface. The aim of this randomised control study was to compare the success of trainees performing a simulated central venous catheter insertion on a phantom either with or without prior NeedleTrainer device practice.

Methods

West of Scotland junior trainees who had not performed insertion of a central venous catheter were randomised into two groups (n=20). Participants undertook standardized online training through a pre-recorded video and training on how to handle a US probe. Group 1 had 10 minutes of supervised training with the NeedleTrainer device. Group 2 were a control group. Participants were assessed on needle insertion to a pre-defined target vein in a phantom. The outcome measures were the time taken for needle placement (secs), number of needle passes (n), operator confidence (0-10), assessor confidence (0-10), and NASA task load index score.

Results

The mean mental demand score in the control group was 7.65 (SD 3.5) compared to 12.8 (SD 2.2, p=0.005) in the NeedleTrainer group. There was no statistical difference between the groups in any of the other outcome measures.

Discussion

This was a small pilot study, and small participant numbers may have impacted the statistical significance. There is natural variation of skill within participants that could not have been controlled for. The difference in pressure needed using the NeedleTrainer compared to a real needle may impact the outcome measures.

## Introduction

Central venous catheter (CVC) insertion is associated with potential complications [1], many of which are linked to the experience of the practitioner [2]. Procedural experience is typically gained during supervised clinical practice and is dependent upon both the quality of training and sufficient case numbers [3,4]. The use of ultrasound (US) guidance has been shown to reduce complications and increase success [5]. However, it requires the development of additional technical skills, including image acquisition and interpretation as well as needle insertion and visualisation.

Simulation allows trainees to practice US-guided CVC insertion in a controlled environment, without the risk of patient harm [5]. NeedleTrainer^TM ^(Intelligent Ultrasound, Cardiff, UK) is a novel device that incorporates a blunt, retractable needle to allow simulation of a procedure on a volunteer, and then augmented reality technology is utilised to superimpose a digital holographic image of the needle trajectory on a real-time US image of the volunteer subject. Unlike practising probe coordination and needling skills on a phantom, NeedleTrainer^ ^allows simultaneous scanning and practice of needle manipulation on human subjects without the need to puncture the skin and without any risk of complications [6]. It is intended for simulation, teaching, and demonstration purposes, though no studies have yet evaluated whether training with the equipment improves CVC insertion, peripheral IV, or nerve block skills. Prior to detailed validation studies, we undertook an exploratory study to investigate whether a period of practice with NeedleTrainer on a low-fidelity phantom model could improve needling skills in novice trainees.

The aim of this study was to compare rates of success in cannulating the internal jugular vein on a phantom in trainees who had received a short period of training using the NeedleTrainer compared with those who had not. Our hypothesis was that practice with the NeedleTrainer device would improve cannulation time, operator confidence, and needle visualisation skills.

## Materials and methods

The West of Scotland Research Ethics Committee confirmed that this study did not require ethical approval. There were no identified safety concerns for participants.

Study population and randomisation

From January 2021 to April 2021, we recruited 20 foundation trainees from the West of Scotland Medical Training Deanery who had no experience of CVC insertion in patients, nor of simulation-based US-guided CVC insertion. Two participants failed to attend the session.

**Figure 1:**
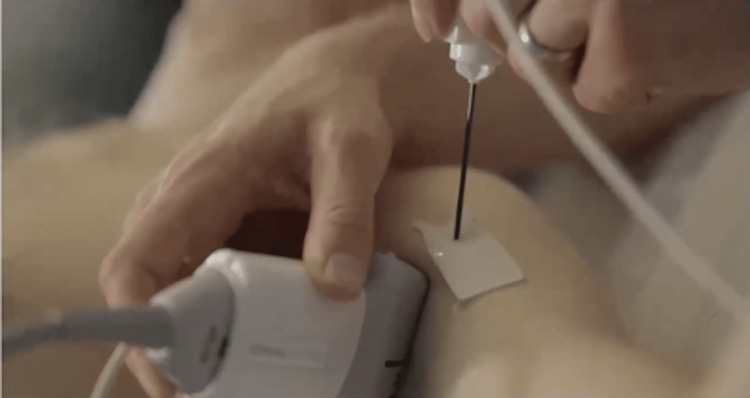
NeedleTrainer Device Image from Ultrasound I. NeedleTrainer - Intelligent Ultrasound [6] NeedleTrainer: Intelligent Ultrasound, Cardiff, UK

Participants were randomised, using a pre-determined online random sequence generator in blocks of 10, to a control or simulation group. The control group (n = 9) received standard teaching on US-guided CVC insertion, and the simulation group (n = 9) received additional training using NeedleTrainer^TM ^(Figure 1). Allocation concealment was achieved using sealed opaque envelopes. Details of the training received by both groups are outlined below.

Study protocol

**Figure 2:**
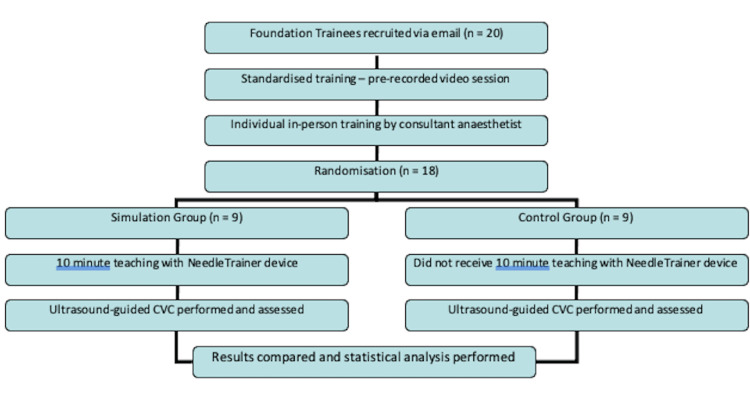
Study flow for all participants CVC: Central venous catheter

The study flow for all participants is shown in Figure 2.

Step 1. Online and In-Person Training for All Participants

All participants watched a pre-recorded video which discussed probe handling; image optimisation; ultrasound physics relevant to needle visualisation; basic needle insertion and visualisation techniques; and the relevant endpoints in this study as well as anatomy and sonoanatomy for CVC insertion in humans. The video also included a demonstration of needling on the phantom by a consultant anaesthetist, emphasising the aim to visualise the needle tip at all times. 

All participants subsequently undertook a time-limited (up to 10 minutes) in-person, one-to-one training session delivered by a consultant anaesthetist (AJRM). This covered the use of the SII ultrasound machine (Sonosite, Bothell, WA), how to hold the 15MHz linear high-frequency ultrasound probe, and how to generate and optimise an image of a vein in short axis on a phantom model (Blue Phantom, Kirkland, WA). The 16G CVC needle and syringe were shown, and trainees were shown how to hold this but were not allowed to practice needle insertion.

Step 2. Simulation-Based Training in the Simulation Group 

Trainees randomised to the NeedleTrainer group had a further time-limited (up to 10 minutes) period of one-to-one training from the same consultant anaesthetist (AJRM). Trainees scanned the same phantom model but now practised a simulated out-of-plane needle insertion with NeedleTrainer. No quantitative endpoint was set during this teaching, but the goal of the session was to practice needle insertion and manipulation to target the vein inside the phantom, learning to keep the simulated needle tip in view. Participants received individualised verbal feedback during this session and, if comfortable, the trainee could terminate the session early. The control group did not receive any time with the NeedleTrainer device.

Step 3. Assessment of CVC Insertion

Each participant performed a US-guided, out-of-plane internal jugular venous puncture on a head and neck torso phantom (Blue Phantom, Kirkland, WA). The primary outcome was the time taken (seconds) to successfully puncture and aspirate fluid from the phantom jugular vein. This was timed by a blinded assessor (AE), starting as the needle tip made contact with the phantom and ending when the participant achieved flashback of fluid from the simulated vein into the needle.

Secondary outcomes included the number of needle passes; expert observer assessment of accuracy of simulated needle placement; operator confidence in accuracy of simulated needle placement; and operator subjective assessment of cognitive load (NASA task load index score). 

The number of needle passes required was counted by a blinded assessor (AE). A needle pass was defined as a change in direction or a withdrawal of the needle. A separate blinded expert assessor (RJK) observed all participants’ performance and scored needle placement on a numeric (0-10) analogue scale [7]. Each participant recorded their confidence in performing the task, also using a numerical (0-10) analogue scale. Participants were also asked to complete a NASA task load index form, which included scoring between 0 and 20 on a numerical analogue scale for each of the following outcomes: mental demand; physical demand; temporal demand; performance; effort and frustration [8].

Statistical analysis

No previous studies had been undertaken with NeedleTrainer upon which to base a power calculation. Statistical analyses were performed using Microsoft Excel (Microsoft Office 2013, Washington, US). The Mann-Whitney U test was used to analyse all outcomes. Statistical significance was defined as p ≤0.05 and p values are reported to two decimal places.

## Results

Eighteen foundation trainees undertook the study. All participants performed a central venous puncture when assessed.

There was no statistically significant difference between groups in the primary outcome of time taken to perform a central venous puncture. The median time taken to reach the vein was 50.5 seconds (IQR 14.0-78.0) in the control group and 69 seconds (IQR 34.0-93.5) in the simulation group (Table 1). No statistically significant difference was noted between groups for the number of needle passes, expert observer score or participant confidence score (Table 1).

**Table 1:** Outcome data IQR: Interquartile range

Outcomes	Simulation Group (median (IQR))	Control Group (median (IQR))	P value
Time Taken to Reach Target (secs)	69.0 (34.0-93.5)	50.5 (14.0-78.0)	P=0.24
Number of Needle Passes (n)	3.0 (1.5-3.8)	2.5 (1.0-4.5)	P=0.91
Observer Assessment (0-10)	6.0 (5.3-6.8)	6.5 (5.3-8.5)	P=0.55
Participant Confidence (0-10)	11.75 (9.3-12.8)	11.0 (8.5-14.5)	P=0.98
Mental Demand (0-20)	13.0 (11.6-14.4)	7.5 (4.8-9.9)	P=0.002
Physical Demand (0-20)	7.25 (6.6-8.8)	4.5 (4.0-8.4)	P=0.25
Temporal Demand (0-20)	8.25 (4.3-14.8)	10.25 (4.3-11.6)	P=0.95
Performance (0-20)	11.75 (9.3-14.5)	13.5 (10.5-15.9)	P=0.38
Effort (0-20)	12.0 (10.5-13.0)	8.75 (6.3-11.9)	P=0.09
Frustration (0-20)	5.5 (3.1-7.8)	4.25 (1.9-11.5)	P=0.93

The only statistically significant difference between groups was noted in the NASA Task Load Index. The median mental demand score in the control group was 7.5 (IQR 4.8-9.9) compared to 13 (IQR 11.6-14.4) in the NeedleTrainer group (p < 0.01). None of the other NASA task load index outcomes were statistically significant (Table 1).

## Discussion

This randomised study found no evidence of difference in the number of needle passes, time taken to reach the target, or either participant or observer confidence between the simulation group and the control group. Patients in the simulation group had higher scores in the domain of mental demand. These data do not support the use of short 10-minute time periods of training using NeedleTrainer for CVC insertion simulation training. The efficacy of training using the NeedleTrainer device for longer time periods should be evaluated in further studies.

Many novice healthcare practitioners feel uncomfortable when performing a CVC insertion procedure for the first time [2]. This feeling of discomfort decreases with clinical experience and the number of times the procedure has been performed with supervision. In an American study of 17 novice trainees, simulation improved both their performance and comfort levels in performing CVC insertion compared to those who had not received simulation training [9]. One possibility for the lack of observed difference in our study may relate to the short ten-minute time period of training, and further studies should include an increased length of training time or a more specific endpoint to suggest skill acquisition. The only between-group difference in our study was a subjectively increased mental demand score following NeedleTrainer simulation training. It is possible that the additional training in the simulation group may have resulted in an increased cognitive load relating to the technology being used, hence the lack of any difference. Similarly, it is possible that participants felt under greater pressure to succeed having received additional training. No qualitative questions were included and so reasons for our findings can only be considered as hypothesis generating.

Simulation offers a cost-effective, standardised, and repeatable method of teaching in a controlled environment at convenient, pre-determined times, and without risk of patient harm [10]. It provides a mechanism for active learning and the ability to receive individualised intra-experience feedback and can support and enhance the development of basic or more advanced technical skills and capabilities at all levels of experience [11]. It is now a component of many training curriculae, including the Royal College of Anaesthetists, with the Joint Royal Colleges and Physicians Training Board (JRCPTB) internal Medicine curriculum stating that ‘all practical procedures in the Internal Medicine stage 1 curriculum should be taught by simulation as early as possible’ [9,10,12,13].

High-quality CVP phantoms contain anatomically correct vasculatures such as the internal jugular vein and carotid artery, the subclavian vein and artery and the superior vena cava. CVC simulation training has been shown to significantly improve success rates of both first cannulation and overall CVC insertion [10]. It is also possible to demonstrate mechanical complications associated with CVC insertion including arterial puncture and pneumothorax [14]. Nevertheless, high-fidelity phantoms do not fully replicate both real-life US scanning and needling. The novel NeedleTrainer allows the operator to scan real human volunteer necks (or any other structure visible with US) and subsequently practice ‘inserting’ a blunt retractile needle into or towards any chosen target. The system does not provide the same tactile feedback as a real-life needle insertion, but the holographic needle image allows the operator to learn the movements required to direct the needle towards the target in real human volunteers. This in theory could help trainees develop the spatial awareness and hand-eye coordination skills required for any US-guided needling procedure including guiding a needle into a central vein, with the advantage of scanning human volunteers rather than even the highest quality phantom.

Our study was randomised, observer-blinded and included a homogenous group of novice practitioners with no prior CVC insertion training or skills. It is the first reported study investigating the NeedleTrainer device for CVC insertion. General training was standardised between groups and delivered by an experienced expert in the use of US for CVC insertion. Limitations of this study includes its small size, the subjective nature of some of the outcomes, and the lack of qualitative experiential data to provide further information on participant experience. This was a small study and no previous studies have been undertaken with the NeedleTrainer device upon which to base a power calculation. Other studies which assessed the Imperial College Surgical Assessment Device (ICSAD) model for quantitative assessment of regional anaesthesia recruited between 20 and 30 patients in total [7,15]. Whilst a Type 2 error remains possible, there was no suggestion of a trend towards significance in outcomes. The time period of training using the Neeedletrainer was short and may have been insufficient to provide adequate skill acquisition. This should be addressed in future studies.

## Conclusions

Although simulation technology can improve training and procedural skills in both novices and more experienced doctors, this small exploratory randomised study in foundation medical trainees found that additional training with the novel NeedleTrainer made no significant difference to CVC insertion on a phantom model other than increasing mental demand. Further larger studies assessing the optimal time period required for training and including volunteers rather than phantoms are required to investigate the potential benefit of the NeedleTrainer device both for central venous access and other US-guided needling procedures.
